# Evolutionary Constraints Acting on DDX3X Protein Potentially Interferes with Rev-Mediated Nuclear Export of HIV-1 RNA

**DOI:** 10.1371/journal.pone.0009613

**Published:** 2010-03-15

**Authors:** Deepak Sharma, Jayanta Bhattacharya

**Affiliations:** Department of Molecular Virology, National AIDS Research Institute, Indian Council of Medical Research, Bhosari, Pune, India; University of California San Diego, United States of America

## Abstract

Differential host-pathogen interactions direct viral replication in infected cells. In HIV-1 infected cells, nuclear export of viral RNA transcripts into cellular cytoplasm is governed by interaction of HIV-1 Rev, Exportin-1 (CRM-1) and DDX3X. Knock down of DDX3X has been shown to drastically impair HIV replication. Here we show that evolutionary forces are responsible for demarking previously unidentified critical functionally important residues on the surface of DDX3X. Using computational approaches, we show that these functional residues, depending on their location, are capable of regulating ATPase and RNA helicase functions of DDX3X. The potential of these residues in designing better blockers against HIV-1 replication was also assessed. Also, using stepwise docking simulations, we could identify DDX3X-CRM-1 interface and its critical functional residues. Our data would help explain the role of DDX3X in HIV-1 Rev function with potential to design new intervention strategies against HIV-1 replication.

## Introduction

**Figure 1:**
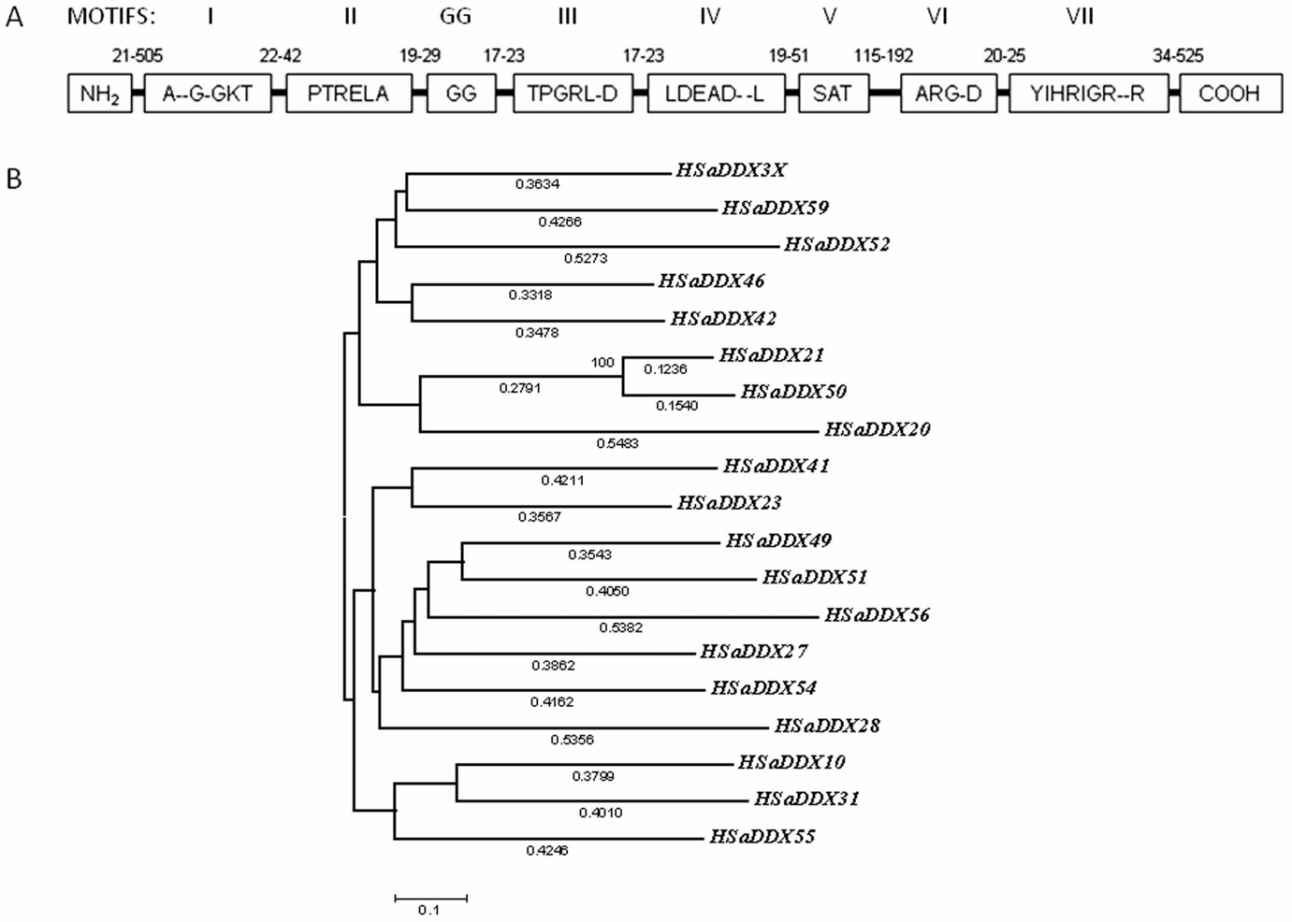
Shortlisted DEAD box helicases after alignment based iterations. A) Conserved DEAD box motifs common to all DEAD box helicases including DDX3X with inter-motif distances. B) Neighbour joining tree for selected DEAD box members. The tree had sum of branch lengths = 8.2995. Cut off for bootstrap test (1000 replicates) = >50%. Branch lengths were shown at the bottom of each branch.

HIV-1 gene expression is regulated by complex interplay of cellular and viral factors. One such important step is posttranscriptional regulation of HIV-1 gene expression by HIV-1 Rev-host factors interaction [1]. Rev binds to a highly structured element called Rev Response Element (RRE) present in all viral transcripts [2]. With the aid of CRM-1/Exportin-1 Rev takes the bound viral RNA transcripts out of the nucleus [3]. In doing so, Rev uses the CRM-1 dependent nucleo-cytoplasmic transport pathway normally associated for export of various proteins in the cell [4]. This pathway employs plethora of host factors. The relation of these host factors in Rev-CRM-1 mediated export of nascent HIV-1 transcripts is not clear. One of the recent findings has shown direct physical interaction of an energy dependent DEAD box RNA helicase DDX3X with expotin-1 (CRM-1) and its implication in HIV-1 Rev function [5]. These helicases are characterized by strong sequence homology with critical motifs encompassing the protein sequence (Figure 1A). One of these motifs is the DEAD box motif and thereby referred to as DEAD box helicases [6]. DDX3X acts as an effector rather than cargo of CRM1/Exportin-1[5]. The actual effector function carried out by this protein is not clear yet, but it seems to regulate HIV-1 Rev mediated RNA export out of the nucleus. Analysis of similar helicases reveals that DDX3X could participate in the terminal step of RNA export by removing proteins that are bound to RNA through nucleopore complex [7]. Recently, DDX3X has also been related to innate antiviral immune mechanisms which highlighted its importance as a molecule being at the crossroads of viral replication and antiviral activity [8]. In this paper, we have applied computational and evolutionary analysis to analyze evolutionary history and to detect putative functional residues that are subject to evolutionary constraints. We then applied this information to protein structure to make the finding functionally more relevant. We identified the pattern of selection inherent in DDX3X at a per site basis by employing maximum likelihood based evolutionary models. Using computational studies and available biochemical data, we predicted the occurrence of specific functionally important residues on DDX3X. These residues have further been shown to be a part of putative binding sites of DDX3X with CRM-1 (exportin-1) as a result of a series of docking simulations. We have also explored the role played by functional residues in affecting different paradigms, such as ATPase function and RNA unwinding of DDX3X function. To the best of our knowledge, our findings gives the first insight about the functional residues of DDX3X which could aid in understanding the route of its action and may help in designing various experimental strategies towards understanding the precise mechanism that HIV-1 Rev employs to export nascent viral RNA transcripts from the nucleus.

## Materials and Methods

### Data Set and Alignment

Primate cluster of sequences was selected from Uniref 90 database [9] (for bias towards human sequences) for each DEAD box family member. Using these clusters, a total of 178 complete nucleotide coding sequences (CDS) belonging to 45 DEAD box family members from 6 different species were retrieved from NCBI Reference Sequence (RefSeq) collection [10]. These sequences were aligned using Gonnet matrix with 3 iterations and divergent cut off delay of 30% (Clustal X) [11]. Subsequent sequence refinement was carried out in MEGA 4 with Clustal W [12] based on two criteria: 1) Average identity between sequences was greater than 50% at the amino acid level and 2) Percentage of gaps was less than 25%. Out of shortlisted members, those with highest pair wise alignment score with respect to DDX3X were finally chosen.

Evolutionary history of the final 19 refined and shortlisted coding sequences (corresponding to amino acid position 167 to 520) was inferred by Neighbour-Joining method [13]. The optimal tree with the sum of branch length = 8.299 was shown. The tree was drawn to scale, with branch lengths (next to the branches) in the same units as those of the evolutionary distances used to infer the phylogenetic tree. The evolutionary distances were computed using the Maximum Composite Likelihood method [14] and were in the units of the number of base substitutions per site. Codon positions included were 1^st^ + 2^nd^ + 3^rd^ + Noncoding. All positions containing gaps and missing data were eliminated from the dataset (complete deletion option). There were a total of 789 positions in the final dataset. Phylogenetic analyses were conducted in MEGA4 [12].

### Selection Analysis

Recombination in the dataset was tested by using a Genetic Algorithm for Recombination Detection (GARD) [15]. Identified breakpoints were assessed by Kishino-Hasegava test implemented in DataMonkey server [16]. To test for diversifying selection and to infer codons under positive selection the ω ratio was calculated with the computer program Codeml from PAML package [17]. The relative fit of codon substitution models was evaluated with likelihood ratio tests (LRT), which were assumed to be χ^2^ distributed with degrees of freedom equal to the difference in number of parameters between models. LRTs for positive selection compare a model in which there is class of sites with ω>1 against a model that does not allow for this class. LRTs were tested with M8 (beta andω) and M8a (beta and ω_s_>1) models. In order to examine the robustness of the positive selections identified by PAML, datasets were also analyzed using two additional methods using HYPHY package [16] (available from DataMonkey facility). These models were single likelihood ancestor counting (SLAC) and fixed effects likelihood (FEL) [18].

For the phylogeny based identification of functionally important residues on DDX3X surface, conservation scores were obtained by likelihood based approach using Jules-Thornton-Taylor (JTT) matrix and using Consurf server (PSI BLAST E-value cut-off = 0.001; Iterations = 3) [19]. The topology based functional residue classification was obtained by structural alignment of identical proteins using Dali facility [20] and subsequent measure of root mean square deviations (RMSD).

### Protein Structures

Protein structure coordinates for DDX3X (2I4I), DDX4 (2DB3), DDX53, DDX47, EIF4A and Exportin-1 (CRM-1) were obtained from RCSB PDB [21]. The structures were energy minimized (steps = 100, step size = 0.2 Å) using rotamers derived from Dunbrack library [22] and the charges were computed using Antechamber method [23]. All the structural modelling was carried out in UCSF Chimera extensible molecular modelling environment [24].

### Estimating Constraints

For the generation of all-atom contact map DDX3X structure was refined by REFMAC, hydrogen atoms were added using REDUCE and contact dots were calculated with PROBE. All these tools were accessed through MolProbity [25]. Docking of ATP molecule to DDX3X was carried using ArgusLab software, version 4.0 [26]. Protein Ligand docking efficiencies had been successfully tested by various groups for ArgusLab [27]. ATP mol2 file was obtained from ChemBank [28]. Hydrogen atoms were added to ligand coordinate file before docking with ArgusLab. Mutant DDX3X structures were generated in Chimera and each structure was minimized. The docking between each mutant DDX3X receptor and ATP was performed by defining a binding site using bound AMP molecule (cubic box = 80×80×80 cubic angstrom). Docking simulations were carried out using “ArgusDock” as docking engine, “Dock” was chosen as the calculation type and AScore was used as the scoring function. All mutant DDX3X-ATP energy results were compared with control docking of wild type DDX3X and ATP. Charge distribution was computed by mapping electrostatic potential (ESP) onto electron density (ArgusLab). This calculation helped to show regions that might act as critical electron acceptor or electron donor. Knowledge based scoring function for protein-ligand interactions implemented in DrugScore was used to analyze individual atomic contributions of functional residues towards ATP binding [29]. Given a protein *I* and a ligand *J*, DrugScore evaluates all nonbonding interactions between protein and ligand atoms to a pseudo energy *ΔW_I,J_* by means of statistically derived atom-type and distance-dependent pair potentials *ΔW_i,j_(r)*:
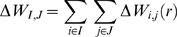



Relative difference in binding energy between mutant and wild- type DDX3X and ATP molecule was obtained from ArgusLab AScore. All the structural alignments were carried out in MatchMaker tool of Chimera and Root Mean Square Deviations (RMSD) were obtained. RNA-DDX3X docking was performed as described above (ArgusLab). DDX3X RNA binding was identified by structural alignment between DDX3X and DDX4 using Chimera. Alanine scanning mutagenesis was performed using Concoord/PBSA [30]. This process mutates structure using WHATIF and a structure ensemble is generated using Concoord. Interaction energies were evaluated by Lennard-Jones potential and Coulomb's Law (parameters from GROMOS96 53a6 force field).

### Pharmacophore Modelling

For ligand based pharmacophore design, multiple flexible alignments of ligands was employed using PharmaGist [31]. The ligand mol2 coordinate files were generated in ArgusLab. Receptor based pharmacophore was defined in FlexX using interaction constraints at ATP binding site of DDX3X [32]. Binding site was identified using bound AMP as an anchor (distance = 10 Å).

### Flexibility Analysis

We performed a Normal Mode Analysis (NMA) on the DDX3X protein structure. We used the approximate NMA developed by Hinsen [33], which represents very well low-frequency domain motions at negligible computational cost. Briefly, a normal mode analysis consists of the diagonalization of the matrix of the second derivatives of the energy with respect to the displacements of the atoms, in mass-weighted coordinates (Hessian matrix). The eigenvectors of the Hessian matrix are the normal modes, and its Eigen values are the squares of the associated frequencies. The low-frequency normal modes are believed to be the ones functionally important. The NMA tools are implemented in the Molecular Modeling Toolkit (MMTK) [34]. All modes were calculated, i.e., three times the number of C*α* atoms (3×418 = 1254). The lowest frequency modes are selected.

Normalized squared atomic displacements (*D*_i_) for each C*α* atom (*i* = 1–994) were calculated as follows:
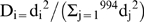
where, **d**_i_ is the component of the eigenvector corresponding to the *i^th^* C*α*.

A vector field representation was calculated as described by Thomas *et al* (34). The vector field was calculated over cubic regions with an edge length of 3 Å, containing on average 1.3 C*α* atoms. The vector field defined on a regular lattice at the center of each cube is the mass-weighted average of the displacements of the atoms in the cube.

### Docking Simulations

We carried out a series of conformation based local and global docking simulations to identify DDX3X-CRM-1 interaction interface. Initial orientations for global docking simulations were carried out in Hex [35] which uses Spherical Polar Fourier (SPF) correlations to accelerate the calculations. Final local docking steps were done in FireDock [36] which predicts the structure of protein complexes given the structures of the individual components and an approximate binding orientation. Both of these programs were tested in CAPRI (Critical Assessment of Predicted Interactions) blind trials and were shown to give accurate results within 1 angstrom of RMSD [37]. Contacts between amino acids were deduced using molprobity. Hydrogen atoms were added using REDUCE and all atom contact map generated by the program PROBE of MOLPROBITY. Amino acid side chain volume was obtained by Voronoi Polyhedra [38].

### Statistical Analysis

All the statistical analyses were carried out in Microsoft Excel software and using standard statistical tables. The alignments were represented using WebLogo [39], [40]. Sets of data were analyzed using Student's t-test. Statistical significance was assessed using P-value: P<0.05, P<0.01 and P<0.001. Standard errors were obtained for all the observations.

## Results

### Identification of DEAD Box Members for Analysis

We acquired 178 nucleotide coding sequences (CDS) corresponding to DEAD box family members (primate cluster) of UniRef. Initial alignment revealed that genes were too divergent for analysis of positive selection. So, the sequences with high number of InDels (Insertion-Deletion) were removed. In addition to that, regions with high diversity were also deleted from all the sequences. DEAD box amino acid region 167 to 520 was finally obtained from remaining 57 CDS. Out of these 57 sequences, those with highest pair wise alignment score with DDX3X coding sequence were kept for further study and rest were discarded. To further reduce any similarity based bias, 19 DEAD box sequences belonging to humans were finalized out of 57 members. These members were represented in a phylogram generated by Neighbour Joining method using amino acids from translated coding sequences (Figure 1B).

### Selection Analysis and Identification of Functionally Important Residues

**Figure 2:**
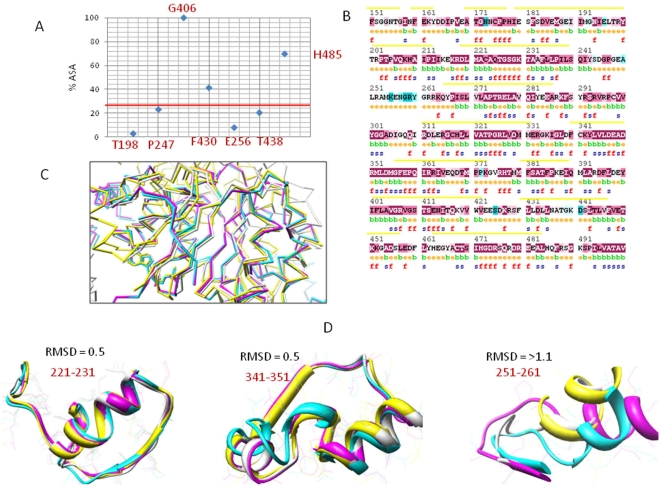
Functional residue identification on DDX3X. A) Positively selected residues plotted against Accessible Surface Area (ASA) B) Residues under negative selection (yellow line) grouped into functional amino acids based on conservation scores. The numbering corresponds to amino acid positions in DDX3X. f: functional residues; s: structural residues; e: exposed; b: buried C) Graph between Root Mean Square Deviation (RMSD) and residue position represents structural alignment of DEAD box members. D) DDX3X structurally aligned with DEAD box helicases.

We aimed to study selection pattern in the selected 167–520 amino acid region of DEAD box members. However, due to the effects of recombination, there is always a probability of obtaining false positive results in any selection study. In order to negate this effect, we first analyzed the recombination acting in these sequences. Recombination analysis revealed the presence of three break points in sequences which could alter the outcome of selection studies. These breakpoints were present at nucleotide positions 186, 769 and 1122 respectively (Figure S1A). We used these breakpoints to generate four separate neighbour-joining (NJ) trees (Figure S1B). Using these trees as an input, four separate selection analysis were carried out. Maximum Likelihood based pair wise comparison of null and alternate hypothesis using a pair of evolutionary models (M0 vs M1 and M8 vs M8a) revealed a log likelihood ratio difference in favour of the alternate hypothesis (2ΔL = 10.4 at 0.005 level of significance). In other words, the presence of positive selection had been indicated in this comparison. Next, we identified sites with significant ratio of dN (non synonymous changes) to dS (dN/dS >1). As shown in Table 1, 10 sites were found to be under positive selection. In addition to positive selection, 158 sites were identified to be under strong purifying or negative selection (Figure 2B). To assess the robustness of positive selection, SLAC and FEL analyses were also carried out (for details, see material and methods). Only the sites which were common in all the four methods were selected as being positively selected (Total 7). It appeared that DDX3X is under strong purifying selection (as inferred from negative selection) with few regions showing evidence for positive selection.

**Table 1:** Selection analyses of DDX3X using maximum likelihood approach.

MODEL	lognL	Parameter estimates	2ΔL	Positively Selected Sites
M8 (Beta and ω)	−19819.6	κ: 1.27707: α: 0. 63411 β: 1.27467 additional omega category: 1.5 prob (additional omega category): 0.0172209	10.4 (0.005)	**T198**, S244, **P247**, A253, F402, **G406**, S410, **F430**, T438, H485, S520
M8a (Null)	−19824.6	κ: 1.23607: α: 0.710644 B: 1.63009 additional omega category set to 1 prob (additional omega category): 0.0699265		NOT ALLOWED
SLAC	------	---------------	N/A (0.2)	**T198, P247, E256**, R294, P297, I310, C317, K335, S382, **G406, F430, T438**, A467, H485,
FEL	------	---------------	N/A (0.2)	**T198, P247, E256, G406, F430, T438**, D455, E458,H85

Next, we used the selection information to highlight functional residues on DDX3X protein. By the term functionally important residues, we mean the residues required by the protein to perform its molecular function or biological role in such a way that they cannot be changed without affecting the function of protein. We identified functional residues based on two criteria:

1. Residues under positive selection and present on surface
2. Surface residues under negative selection with inferred functional importance

For identifying functional residues with positive selection, we simply mapped the positively selected sites on DDX3X and related them with Accessible surface area (ASA) (Figure 2A). Sites with accessible area greater than 30% were identified as functional. Out of the identified positively selected sites, Gly^406^, Phe^430^, and His^485^ were identified as functional residues.

For short listing functional importance in negatively selected amino acids, we employed a phylogenetic sequence based method profiling of DEAD box helicases. The alignment of selected DEAD box helicases was used to generate a residue wise conservation score using maximum likelihood approach (Jules-Thornton-Taylor matrix). The conservation score corresponds to evolutionary rate of that position. Positions were colour coded according to these scores and are depicted in Figure 2B. Only those residues were selected as functional which show high conservation scores along with surface accessibility of greater than 30%. These functional residues identified by sequence based phylogenetic approach were further validated using an independent approach based on structure comparisons. Pair wise structural alignment between DDX3X and other members of DEAD box helicase family revealed that the Root Mean Square Deviations (RMSD) at the functional residue sites identified by sequence based approach, was least while the RMSD at the non functional sites was maximum (Figure 2C and 2D).

### Role of Functional Residues in Generating Constraints at ATP Binding Site of DDX3X

ATP binding to DDX3X was utilized as a potential target where a block was shown to result in inhibition of HIV-1 Rev activity [41]. ATP analogs, which bind to ATP binding site of DDX3X, were shown to inhibit HIV-1 replication [42]. We thought that identification and analysis of critical functional residues at this site would aid in explaining the specificity of DDX3X- ATP binding site and designing of selective and efficient inhibitors that could result in inhibition of HIV-1 replication. So, we studied the constraints acting at this site of DDX3X with reference to functional residues and analyzed their selectivity towards DDX3X.

**Figure 3:**
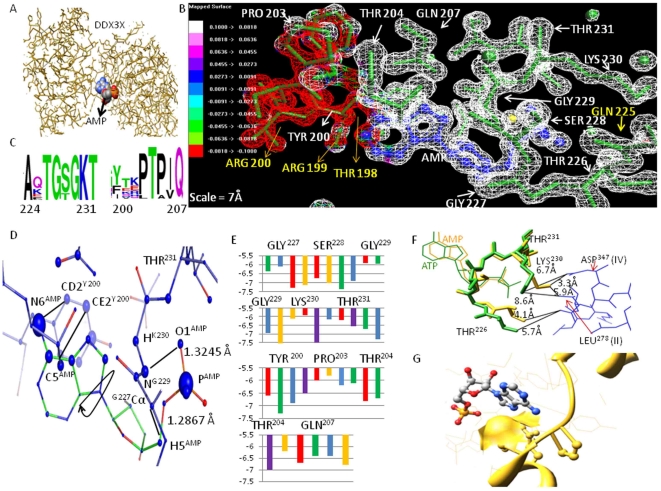
Functional residues at the ATP binding site of DDX3X. A) DDX3X structure (wireframe) showing the bound AMP (sphere) at the groove formed by bilobed DDX3X. B) Electrostatic potential map showing charge distribution near essential functional residues. Red are regions of negative ESP (Electrostaic potential). White represents positive ESP C) Alignment showing entropy at each functional residue position with most conserved residues having highest bit score. D) Atomic contributions for ATP binding for functional residues. Atoms contributing maximum are shown with spheres of large radius. The inter-atomic distance are shown alongside critical ATP-DDX3X association and is given in the units of Angstroms. E) Binding energy change values on substitutions at various functional residue positions. Substitutions: red – with positive charged residues; green – with negative charged residues; blue – with polar residues; yellow – with aromatic residues and purple – with non polar residues. F) Atomic displacements on ATP-AMP conversion. Thr226, Lys230 and Thr231 occupy critical positions in contact with residues of motifs II and IV. G) Q207 and P204 occupied crucial positions in the ATP binding site of DDX3X. Q207 forms the base of this cavity while P204 helps in alpha helix torsion angle.

We started by short listing the functional residues that formed DDX3X-ATP binding site. We found that ATP binds in a groove formed due to the bi-lobed nature of DDX3X protein structure (Figure 3A). Using DDX3X crystal structure (PDB ID: 2I4I), an all atom contact map was created and AMP was selected as a marker point (AMP was present in the bound form in DDX3X crystal structure). Amino acids in the radius of 7 Å of AMP (corresponding to distance up to which inter atomic van der Wall forces are felt) were taken as members of its binding site. This selection revealed residue positions 200–207 and 225–231 in the proximity of AMP with Tyr^200^, Pro^203^, Trp^204^, Gln^207^, Gln^225^, Thr^226^, Gly^227^, Ser^228^, Gly^229^, Lys^230^ and Thr^231^ being the functional residues (based on conservation score). ATP molecule was docked to DDX3X structure and the structure was energy minimized. We utilized inter-atomic interaction information for the identified functional residues to study constraints acting on them. Electrostatic potential-mapped electron density surface [39] with inter-atomic contacts revealed that residues 227–231 formed close and direct inter-atomic associations (inter-atomic distance between 1–3 Å) with phosphate and ribose moieties of bound ATP molecule (ATP tail) with a preponderance of net positive charge (Positive ESP or Electrostatic Potential) (Figure 3B). Also, these residues were found to be highly conserved among DEAD box members (Figure 3C). Gly^227^ and Gly^229^ atomic contributions were found to be maximum towards binding ATP tail, with their Cα and N atoms in close contact with ATP H-5 and O-1 atoms (1.3245 Å and 1.2867 Å) respectively (Figure 3D). Substitutions with positively charged and large polar aromatic residues were found to substantially reduce ATP-DDX3X binding energy (Figure 3E). On the other hand, Lys^230^ hydrogen atoms were in close proximity to phosphate group of ATP (ATP tail) (with inter-atomic distances = 2.0965 Å–3.6543). These three functional residues thus carry greater share of ATP-DDX3X binding energy with strong positive charge responsible for stabilizing ATP tail region (Figure 3B and 3D). Also, in association with Thr^226^ and Thr^231^, Lys^230^ makes important inter-domain contacts with motifs which participate in RNA unwinding process of DDX3X (Motifs II and IV) (Figure 3F). Probably, because of this direct role in ATPase activity and indirect role in RNA unwinding mechanism, Thr^226^, Thr^231^ and Lys^230^ were found to be less tolerant towards any change (average ΔBE >2 kcal/mol). Among other functional residues, Tyr^200^ near the purine ring of bound ATP molecule was quite tolerant towards substitutions, which could be attributed to the ability of AMP purine ring to adopt different poses on binding DDX3X (Figure 3D). Pro^203^ was another interesting functional residue found to maintain DDX3X-ATP binding site (probably because of its function of extension of otherwise α helical segment of the ATP binding cleft) with significant effect on ATP binding energies (Figure 3E). Thr^204^ and Gln^207^ occupied interface of positive and negative charge distribution of the ATP binding cleft (Figure 3B) and were responsible for forming its (ATP binding cleft) base (Figure 3G). They were highly conserved among DEAD box members and most of the size changing variations here were damaging for ATP binding (because of changes in ATP binding cavity area). So, taken together, we found that depending on the contribution towards ATP binding and depending on the role in maintaining the structural integrity of the ATP binding site, different functional residues at the DDX3X-ATP binding site were under various constraints which maintain their sequence conservation. In addition to the functional residues described above, we performed a DDX3X specific functional residue identification and found Thr^198^, Arg^199^, Arg^202^ and Gln^225^ to be specific to DDX3X (Figure 3B).

**Figure 4:**
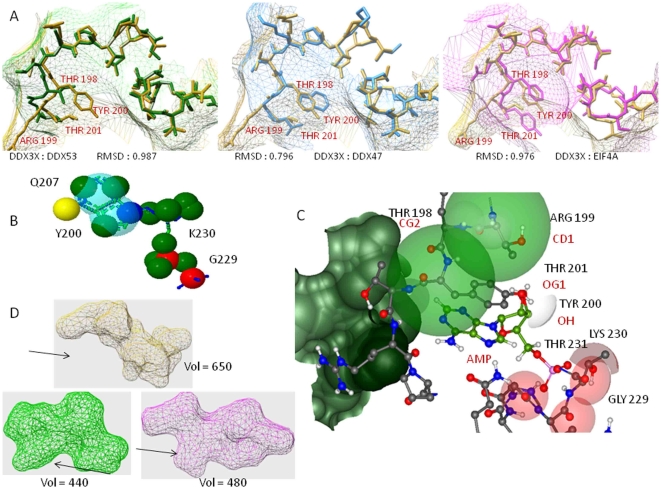
Comparison of ATP binding site of DDX3X with other helicases. A) Structural alignments of DDX3X with DDX53, DDX47 and EIF4A showing variations at residue positions 198–201. B) Pharmacophore designed using known AMP analogs with electron donors and electron acceptors highlighted. C) Receptor based pharmacophore based on combination of conserved and DDX3X specific functional residues. Atoms selected (donors and acceptors) were shown beneath each residues. Hydrogen donors: White, Hydrogen acceptors: Red, Aromatic: Green. D) Conformations of the DDX3X specific residues at position 198 to 201. Corresponding residues in other DEAD box helicases mainly yield two kinds of surfaces as shown. DDX3X surface has larger binding cavity at this site which was not found in any other helicase.

Next, we examined whether the functional residue constraints were responsible for generating similar architecture of ATP binding site for all DEAD box members or protein specific variations exist. We used crystal structures of available DEAD box helicases and compared their ATP binding regions, encompassing the functional residues shown in Figure 3B, with DDX3X ATP binding site. As shown in Figure 4A, the root mean square deviations (RMSD) of structural alignment between these helicases were less than 1 Å, indicating a predominantly similar arrangement of functional residues. However, the positions Thr^198^, Arg^199^, Tyr^200^ and Thr^201^ showed variations among all the studied structural alignments. In addition, these residues showed specific structural arrangement, exclusive to DDX3X, which was not seen in any other helicase. Surface volume measurements (Figure 4A, mesh surface) at these residue positions showed the depth of DDX3X-ATP binding cavity to be more compared to other helicases (average Δ vol = 58 Å).

The AMP analogs were shown by various groups as efficient blockers of DDX3X ATPase activity (and HIV-1 replication) [39], [40]. We carried out a multiple flexible alignment of all the AMP analogs in order to analyze their stability and selectivity, with respect to the constraints that we identified in Figure 3 and Figure4. The aligned analogs were employed for the generation of a pharmacophore (i.e. corresponding binding site on DDX3X). The pharmacophore, as shown in Figure 4B revealed that these analogs mainly utilize the functional residues which are under structural constraints in all the DEAD box helicases. Because of this, these analogs can bind to most of the helicases and, thus possess minimum selectivity. In fact, we docked the pivot molecule (AMMPNP which is a nonhydrolyzable analog) to different DEAD box helicases and found that, with small variations in ligand-DDX3X binding energy values, the ligand was capable of binding all helicases. We thought that to improve the DDX3X specific selectivity of analogs, residue positions with high atomic contributions towards ATP binding (Figure 3D) can be combined with DDX3X specific functional residues that were shown in Figure 4A. We used residue positions 227 to 231 and residue positions 198 to 201 to design a novel pharmacophore specific to DDX3X (Figure 4C). The residues 227-231 could serve to provide ample binding strength, because of their maximum contributions towards ATP-DDX3X binding while residues 198 to 201 could provide selectivity towards DDX3X (Figure 4D).

### Role of Functional Residues in Regulating RNA Unwinding Function of DDX3X

**Figure 5:**
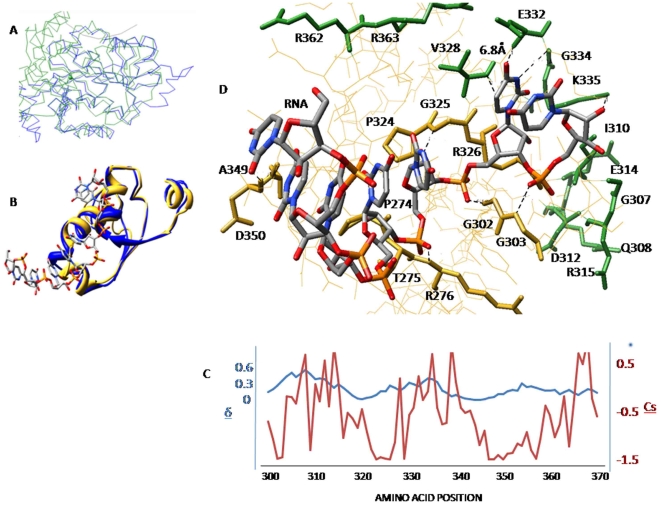
Functional residues at the RNA binding site. A) Structural alignment of C alpha traces of DDX3X and DDX4. B) RNA binding region of DDX3X structurally alignment with DDX4 (RMSD = 0.4). C) Plot of normalized C alpha displacements with conservation scores to identify flexibility of functional residues at this region. D) Flexible (green) and rigid (yellow) functional residues identified at the RNA binding region of DDX3X.

**Figure 6:**
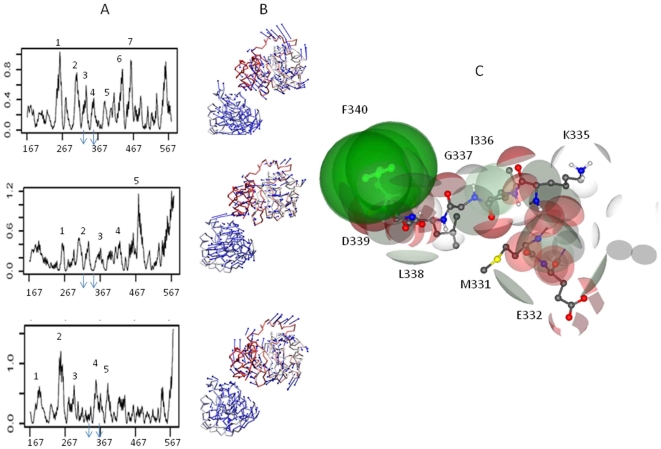
Role of functional residues at RNA binding site of DDX3X. A) Normal mode analysis of DDX3X with modes having lowest deformation energy values selected. Vector fields representation to show the residues wise displacement in DDX3X. B) Cavity identification at the RNA binding region and selection of potential electron donors and acceptors. C) Hydrogen donors: White, Hydrogen acceptors: Red, Aromatic: Green.

Apart from ATP binding site, another important DDX3X surface is the region where RNA binds. Since RNA binding region was not clearly understood for DDX3X, we carried out a structure based alignment using DDX3X structure as query (in CATH database) and identified DDX4 as a DEAD box helicase which shares >70% similarity with DDX3X. This helicase was recently co-crystallized along with bound synthetic RNA molecule [43]. Since the RMSD (Root Mean Square Deviation) of the RNA binding region of DDX4 and the corresponding region of DDX3X was less than 0.5 Å (Figure 5A), we decided to utilize the RNA binding site of DDX4 as a backbone to identify RNA binding region in DDX3X. By homology, we identified DDX3X amino acid region spanning amino acids 300 to 370 as potential RNA binding site (Figure 5B). Docking of the synthetic RNA (from DDX4 structure) to this region of DDX3X using distance constraints (ArgusLab) revealed that DDX3X RNA binding region was mainly composed of an alpha helix (Figure 6B). The functional residues within 7 Å of bound RNA formed this helix and were described in Figure 5D. We carried out computational alanine scanning mutations at these regions and identified the binding “hot spots” among the functional residues by obtaining the binding energy change values (ΔΔG). Functional residues that showed ΔΔG >2kcal/mol after alanine substitutions were considered important for RNA-DDX3X interaction and are shown in Figure 5D. These residues were part of the conserved DEAD box motifs II, III, IV and V (Fig 1A). In addition to binding strength between RNA and DDX3X, this region also requires certain level of conformational rearrangement and flexibility in order to accomplish the process of RNA unwinding. We decided to analyse the role of functional residues in providing flexibility to this RNA binding region of DDX3X. For this, we started by estimating the flexibility at this region, as a whole, and then used this information to highlight important functional residues showing maximum atomic displacements. We used, NMA of DDX3X which is an approach based on the hypothesis that vibrational normal modes of a protein exhibiting the lowest frequencies (also named soft modes) describe the largest movements in a protein and are the ones functionally relevant (for details see Materials and Methods). Each normal mode represented a combination of conformational displacements achieved by amino acid residues in a protein. Normal modes were generated by MMTK package using Cα atoms of each residue as representative of the masses of whole amino acid. Out of 222 calculated DDX3X normal modes (each mode represents protein motion), we selected mode numbers 7-9, because these modes represented the lowest deformation energy values (low deformation energy means a mode with large rigid regions and a good chance for describing individual domain motions). To analyze displacements associated with these three modes, we calculated normalized square atomic displacement for each mode, which is the square of the displacement of each C*α* atom, normalized, so that the sum over all the DDX3X C*α* atoms equals one. Graphs for all three modes showed peaks for normalized atomic displacements corresponding to RNA binding region of DDX3X thus, indicating that this region was flexible (Figure 6A). Residue wise flexibility was also mapped on the DDX3X structure and was shown in the form of vector field to highlight the direction and amplitude of displacements (Figure 6B). We identified the functional residues corresponding to these peaks and plotted the normalized atomic displacements of each functional residue with conservation score values (Figure 5C). As shown in Figure 5D, the binding “hot spot” functional residues were mostly rigid. As previously discussed, these residues were members of conserved DEAD box motifs II, III, IV and V. On the other hand, the functional flexible residues were found to be separate from the conserved motifs of DEAD box helicases, thus implicating presence of different sets of functional residues for providing DDX3X-RNA binding and for providing flexibility for RNA unwinding respectively.

Comparing the DDX3X RNA binding site from different DEAD box helicases (using approach similar to Figure 4A) showed that this region exhibits better member specific selectivity than the ATP binding region. We thought of utilizing the flexible and rigid residues (from Figure 5D) to identify a potential site which can be used as a target for a blocker molecule. To do that, the RNA binding region was examined for the presence of cavities on the DDX3X surface. A cavity encompassing regions 330 to 345 of RNA binding region was found to include both flexible and rigid flexible residues. This cavity was selected and the nature of residues in this cavity was analyzed by constructing a map of charge distribution, calculated using ArgusLab as shown in Figure 6C. Important electron donors and acceptors belonging to crucial functional residues were highlighted.

### DDX3X-CRM-1 Docking and Functional Effects of Constraints on DDX3X-CRM-1 Interaction

**Figure 7:**
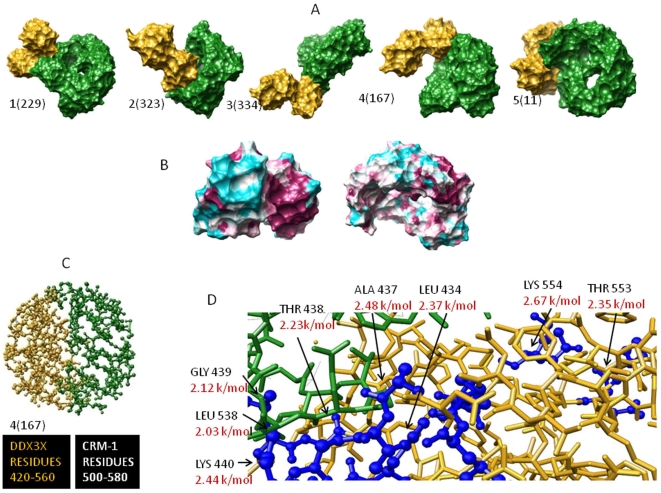
Docking of DDX3X-CRM-1. A) Top 5 docked complexes B) Phylogenetic identification of clusters with binding hot spots. C) Interface of selected complex with identified functional residues. D) Binding energy values are shown in Red.

Another aspect of DDX3X function is its interaction with Exportin-1 (CRM-1) to accomplish efficient transport of newly formed HIV-1 RNA with the help of HIV-1 Rev protein. We wondered whether the functional residues that we found in our study could play any role in DDX3X-CRM-1 interaction (and subsequently in Rev Activity). Since the exact binding region for DDX3X-CRM-1 is not known, we started by docking DDX3X onto CRM-1 using a combination of algorithms based on conformation based matching. Before performing this analysis we checked the accuracy of these docking algorithms using known complexes (data not shown). We realized that our approach would find the interface of a known complex within 1.5 Å of fluctuation (RMSD between experimental and docked complex was <1.5 Å). Using a combination of Geometric Hashing, Pose-Clustering matching and *spherical polar Fourier (SPF)* techniques [35], we generated initial starting positions for proteins compared to each other. For this, Exportin-1 PDB structure (ID: 3GB8) and DDX3X PDB structure (ID: 2I4I) were used (obtained from RCSB PDB). Pre-docking structure edit was done to add hydrogen residues, remove surface water molecules and to correct deviant bond angles and atoms [36]. Molecular centroids of two protein structures were adjusted so that they fall within 30 angstrom radius wall. We used initial steric scan of 20 followed by a final search at N = 30 (other parameters were Distance range = 40, Scan Step =  0.75, Sub steps = 2) [35]. All but top 25000 entries were discarded. Spatially similar orientations were grouped together and clusters were made starting from the solution with highest energy value. Out of these, top 10 clustered orientations are given in Table S1 in order to give an indication of the parameters considered for this analysis. We sought to refine these initial positions generated by rigid body docking algorithms by Monte Carlo minimization of the binding score function [35]. The refined candidates were ranked by the binding score. This score includes Atomic Contact Energy, softened van der Waals interactions, partial electrostatics and additional estimations of the binding free energy [37]. To do this, we utilized top 500 transformation files obtained by global docking approach. Refined candidates were ranked according to their energy function as discussed before and as shown in Table S1. Final refinement was again carried out on the best 60 structures obtained following initial local refinement to reduce the incidence of clashes (using full interface Side-chain optimization). Structures obtained following final local docking were arranged on the basis of their global energy parameters. Out of these complexes, five structures having highest global energy parameters were selected (Figure 7A).

Structure parameters were employed to dock DDX3X to CRM-1. To select one potential complex out of five structures, we utilized an additional phylogenetic approach based on maximum likelihood analysis to highlight clusters of interaction “hot spots” on both DDX3X and CRM-1 surface. Then, we selected the DDX3X-CRM-1 complex (out of final five complexes) that had an interface with maximum concentration of hot spots (Figure 7B). Based on this, we could select structure four, as shown in Figure 7B, to be the possible DDX3X-CRM-1 complex. DDX3X region encompassing amino acid residues 420–560 formed contact with CRM-1 region with amino acids 500–580 (Figure 7C). Most of the potential functional residues under positive selection were present in this region (G406, F430, and H485). Next, we used the residue wise conservation scores (from Figure 3) and selected the functional residues in this region. These functional residues were evaluated for their role in DDX3X-CRM-1 binding by running a carbon probe of 1.4 Å (a carbon probe represents a peptide binding region) on the DDX3X part of the DDX3X-CRM-1 interface. In addition, alanine scan of the functional residues at this interface was also carried out. Finally, functional residues contributing highly towards DDX3X-CRM-1 binding (found by carbon probe-DDX3X functional residue binding energy values) and showing maximum change in binding energy (ΔG as found by alanine scanning) were identified as residues crucial for DDX3X-CRM-1 association and were shown in Figure 7D.

## Discussion

In this paper, we identified critical functional residues regulating ATPase and RNA unwinding function of DDX3X (Figures 3, 4, 5, 6) and predicted DDX3X-CRM-1 interaction region (Figure 7). We believe this information could provide a new and detailed glimpse of the mechanism by which complex interplay of DDX3X-HIV-1 Rev-CRM-1 takes place and leads to efficient viral replication. Using the functional residue information at the DDX3X ATPase site, we showed that most of these residues are under strong functional constraints and that validates their conservation pattern (Figure 3). Using computation based biochemical approaches; we then identified the cause for these constraints as well (Figure 3B, D, E, F, G). This information helped us in comparing the ATP binding sites of different DEAD box helicases (Figure 4A). Most of the functional residues were common to all the sites with little structural variations (Figure 4A). But, certain ATP proximal functional residues showed remarkable specificity for DDX3X. We believe that these regions are responsible for providing different levels of ATPase activities to different DEAD box helicases. The binding energy for ATP binding regions of different DEAD box helicases, when compared, showed substantial variations, mainly attributable to amino acid positions 198 to 201 (the DDX3X specific region). Using ligand based pharmacophore modelling; we showed that most of the available AMP analogs bind residues which were common to all DEAD box helicases (Figure 4B). In fact, docking of these analogs to different DEAD box helicases revealed that they were capable of binding to all of these members with minor variations in binding energy values. We utilized the functional residue information to select region on DDX3X that could be employed for generation of DDX3X specific analog. Surface topology of the selected region showed that this surface had specific conformation specific to DDX3X. Comparing this surface for other helicases revealed a predominant two kind of topologies (Figure 4D) which were different for DDX3X. So, combining surface and electron donor-acceptor profile of the selected region at ATP binding site, we identified a pharmacophore that can be potentially employed to design DDX3X specific ATP analog (Figure 4C).

As found by docking simulations, the binding of DDX3X to CRM-1 positions DDX3X in such a way that its helicase domain was found to come in proximity of HIV-1 Rev, which binds CRM-1 (Figure S2). It indicated that HIV-1 Rev transfers the viral RNA to DDX3X for unwinding during the process of nuclear transport. Also the flexible and rigid functional residues at the RNA binding region indicate their role in RNA unwinding process (Figure 5). The identification of functional residues at this site showed that the flexible and rigid functional residues constitute separate clusters and the highly conserved motifs of the DDX3X encompassed a predominance of rigid residues. This indicates that residues required for maintaining DDX3X dynamics are different from residues required for efficient RNA-DDX3X binding. This information can help in understanding the mechanism of HIV-1 RNA unwinding by DDX3X in more detail. Further, we showed that RNA binding region showed better selectivity and specificity towards DDX3X as compared to the ATP binding region by comparing the structural topology of different DEAD box helicases. We believe that this region can be utilized to generate better selective inhibitors for potential block in HIV-1 replication (Figure 6C).

Analysis of functional residues is crucial for characterization of domains present in a protein. Functional residue information of DDX3X (Figure 2) would provide more information about the mechanism by which this helicase acts and about the selectivity that differentiates DDX3X from rest of the helicases of the family. Direct role of DDX3X in HIV-1 Rev mediated nuclear export of unspliced viral RNA further makes it important to understand the functional sites of DDX3X. Development of potential ligand molecules that block specific DDX3X function could help in achieving the aim of designing ideal intervention against HIV-1 replication.

## Supporting Information

Figure S1 Recombination analysis for the DEAD box helicases. A) The break points represented based on Kishino-Hasegava test at DDX3X codon positions 186, 789 and 1122, respectively. B) Separate neighbour joining trees corresponding to each break point.
(1.03 MB TIF)

Figure S2 Predicted association of DDX3X, CRM-1 and HIV-1 Rev. DDX3X docking with CRM-1 was found to orient DDX3X helicase domain in proximity to HIV-1 Rev that binds CRM-1 at its residue position 800 to 820.
(1.56 MB TIF)

Table S1 Stepwise docking simulations for DDX3X-CRM-1 interaction.
(0.03 MB PDF)

## References

[1] Harris ME, Hope TJ (2000). RNA export: Insights from viral models.. Essays Biochem.

[2] Malim MH, Hauber J, Le SY, Maizel JV, Cullen BR (1989). The HIV-1 rev trans-activator acts through a structured target sequence to activate nuclear export of unspliced viral mRNA.. Nature.

[3] Askjaer P, Jensen TH, Nilsson J, Englmeier L, Kjems J (1998). The specificity of the CRM1-Rev nuclear export signal action is mediated by RanGTP.. J Biol Chem.

[4] Neville M, Stutz F, Lee L, Davis LI, Rosbash M (1997). The importin-beta family member Crm1p bridges the interaction between Rev and the nuclear pore complex during nuclear export.. Curr Biol.

[5] Yedavalli V, Neuveut C, Chi Y-H, Kleiman L, Jeang K-T (2004). Requirement of DDX3X DEAD Box RNA Helicase for HIV-1 Rev-RRE Export Function.. Cell.

[6] Lüking A, Stahl U, Schmidt U (1998). The Protein Family of RNA Helicases.. Critical Rev Biochem Mol Biol.

[7] Rocak S, Linder P (2005). DEAD-box proteins: the driving forces behind RNA metabolism.. Nature Rev Mol Cel Biol.

[8] Soulat D, Burckstummer T, Westermayer S, Goncalves A, Bauch A (2008). The DEAD-box helicase DDX3X is a critical component of the TANK-binding kinase 1-dependent innate immune response.. EMBO J.

[9] Suzek BE, Huang H, McGarvey P, Mazumder R, Wu CH (2007). UniRef: comprehensive and non-redundant UniProt reference clusters.. Bioiformatics.

[10] Pruitt KD, Tatusova T, Maglott DR (2005). NCBI Reference Sequence: a curated non-redundant sequence database of genomes, transcripts and proteins.. Nucl Acid Res.

[11] Thompson JD, Gibson TJ, Plewniak F, Jeanmougin F, Higgins DG (1997). The CLUSTAL_X windows interface: flexible strategies for multiple sequence alignment aided by quality analysis tools.. Nucleic Acids Res.

[12] Kumar S, Tamura K, Nei M (2004). MEGA4: Integrated software for Molecular Evolutionary Genetics Analysis and sequence alignment. Brief.. Bioinform.

[13] Saitou N, Nei M (1987). The neighbor-joining method: a new method for reconstructing phylogenetic trees.. Mol Biol Evol.

[14] Tamura K, Nei M, Kumar S (2004). Prospects for inferring very large phylogenies by using the neighbor-joining method.. Proc Natl Acad Sci (USA).

[15] Pond K, Frost SDW (2005). A Genetic Algorithm approach to detecting lineage specific variation in selection pressure.. Mol Biol Evol.

[16] Pond K, Frost SDW (2005). Datamonkey: rapid detection of selective pressure on individual sites of codon alignments.. Bioinformatics.

[17] Yang Z (1997). PAML: A program package for Phylogenetic analysis by maximum likelihood.. Comput Appl Biosci.

[18] Pond K, Frost SDW (2005). Not so different after all: A comparison of methods for detecting amino acid under selection.. Mol Biol Evol.

[19] Landau M, Mayrose I, Rosenberg Y, Glaser F, Martz E (2005). ConSurf: the projection of evolutionary conservation scores of residues on protein structures.. Nucl Acid Res.

[20] Holm L, Kaariainen S, Rosenstrom P, Schenkel A (2008). Searching protein structure databases with DaliLite V.3.. Bioinformatics.

[21] Berman HM (2000). “The Protein Data Bank”.. Nuc Acid Res.

[22] Dunbrack RL (2002). Rotamer Libraries in the 21^st^ century.. Curr Opin Str Biol.

[23] Wang J, Wang W, Kollman PA, Case DA (2006). Automatic atom type and bond type perception in molecular mechanics calculation.. Journal of molecular graphics and modelling.

[24] Pettersen EF, Goddard TD, Huang CC, Couch GS, Greenblatt DM (2004). UCSF Chimera–a visualization system for exploratory research and analysis.. J Comput Chem.

[25] Davis IW, Murray LW, Richardson JS, Richardson DC (2004). MOLPROBITY: structure validation and all-atom contact analysis for nucleic acids and their complexes.. Nuc Acid Res.

[26] Thompson MA ArgusLab 4.0.1. ArgusLab 4.0.1. Planaria Software LLC, Seattle, WA.. http://www.arguslab.com.

[27] Joy S, Nair P S, Hariharan R, Pillai MR (2006). Detailed comparison of the protein-ligand docking efficiencies of GOLD, a commercial package and ArgusLab, a licensable freeware.. In Silico Biology.

[28] Seiler KP, George GA, Happ MP, Bodycombe NE, Carrinski HA (2008). ChemBank: a small-molecule screening and cheminformatics resource database.. Nuc Acid Res.

[29] Gohlke H. Hendlich M, Klebe G (2000). Knowledge-based scoring-function to predict protein-ligand interactions.. J Mol Biol.

[30] Benedix A, Becker CM, Groot L, Caflisch A, Rainer AB (2009). Predicting free energy changes using structural ensembles.. Nat Met.

[31] Schneidman-Duhovny D, Dror O, Inbar Y, Nussinov R, Wolfson J (2008). PharmaGist: a webserver for ligand-based pharmacophore detection.. Nuc Acid Res.

[32] Rarey M, Kramer B, Lengauer T, Klebe A (1996). Fast Flexible Docking Method using an Incremental Construction Algorithm.. J Mol Biol.

[33] Hinsen K (1998). Analysis of domain motions by approximate normal mode calculations.. Proteins.

[34] Hinsen K (2000). The molecular modelling toolkit: a new approach to molecular simulations, J Comput Chem.

[35] Ritchie DW (2005). High Order Analytic Translation Matrix Elements For Real Space Six-Dimensional Polar Fourier Correlations.. J Appl Cryst.

[36] Andrusier N, Nussinov R, Wolfson HJ (2007). FireDock: Fast Interaction Refinement in Molecular Docking.. Proteins.

[37] Vajda S, Kozakov D (2009). Convergence and Combination of Methods in Protein-Protein Docking.. Curr Opin Str Biol.

[38] Bohm H, Heimann RB, Bohm M (2003). Voronoi Polyhedra: A useful rool to determine the symmetry and bravais class of crystal lattices.. Cryst Strc Symm.

[39] Crooks GE, Hon G, Chandonia JM, Brenner SE (2004). WebLOgo: A sequence logo generator.. Gen Res.

[40] Schneide TD, Stephens RM (1990). Sequence Logos: A new way to display consensus sequences.. Nuc Acid Res.

[41] Yedavalli VS, Zhang N, Cai H, Zhang P, Starost MF (2008). Ring expanded nucleoside analogues inhibit RNA helicase and intracellular human immunodeficiency virus type 1 replication.. J Med Chem.

[42] Maga G, Falchi F, Garbelli A, Belfiore A, Witvrouw M (2008). Pharmacophore modelling and molecular docking led to the discovery of inhibitors of human immunodeficiency virus-1 replication targeting the human cellular aspartic acid-glutamic acid-alanine-aspartic acid box polypeptide 3.. J Med Chem.

[43] Sengoku T, Nureki O, Nakamura A, Kobayashi S, Yokoyama S (2006). Structural Basis for RNA Unwinding by the DEAD-Box Protein Drosophila Vasa.. Cell.

