# Supplementary figures and images for: Evolutionary Constraints Acting on DDX3X Protein Potentially Interferes with Rev-Mediated Nuclear Export of HIV-1 RNA
Source: PLoS One. 2010 Mar 15;5(3):e9613. doi: 10.1371/journal.pone.0009613 (PMC2837722; doi:10.1371/journal.pone.0009613)

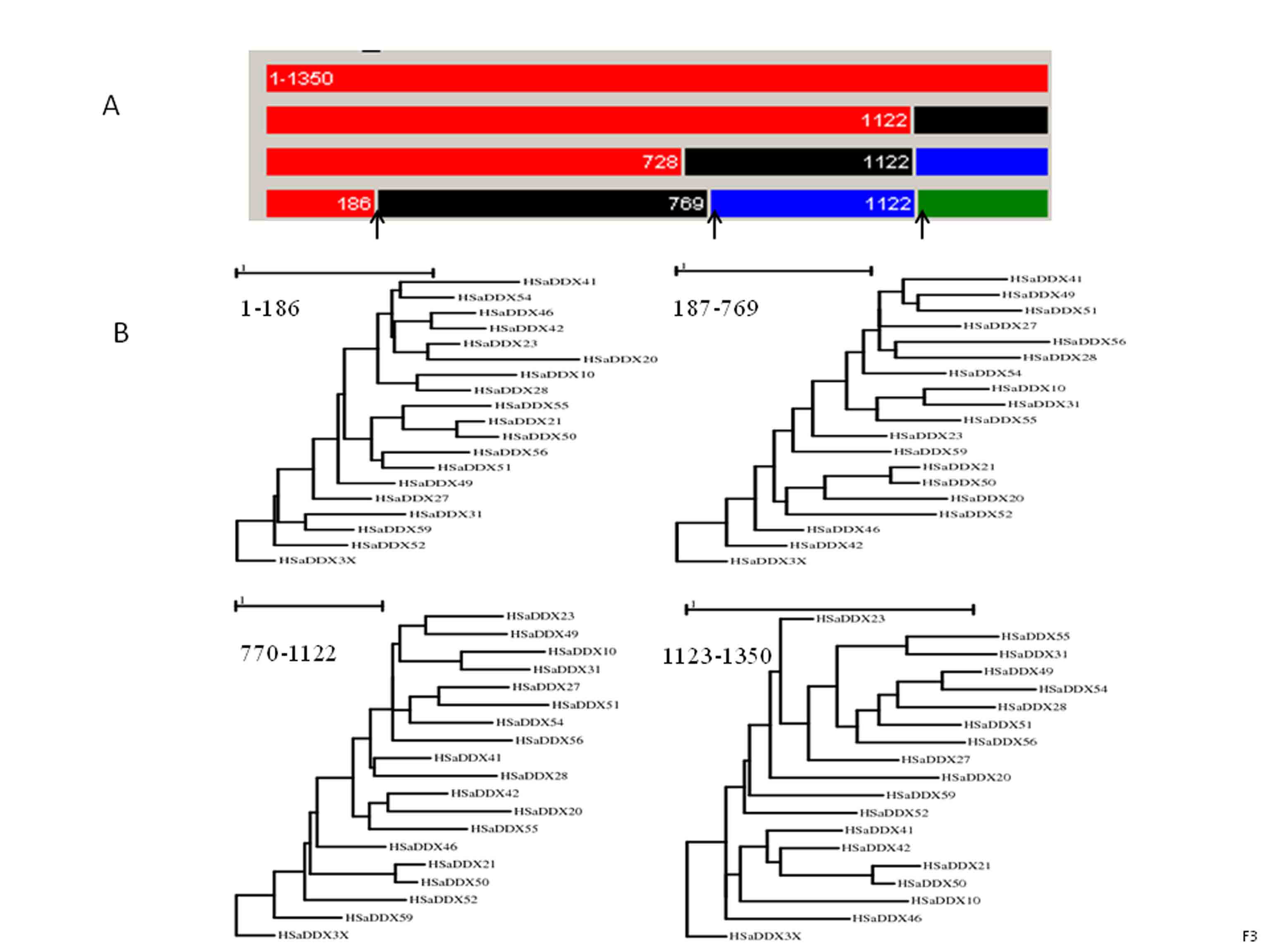

Supplement: Figure S1 — Recombination analysis for the DEAD box helicases. A) The break points represented based on Kishino-Hasegava test at DDX3X codon positions 186, 789 and 1122, respectively. B) Separate neighbour joining trees corresponding to each break point. (1.03 MB TIF) [file pone.0009613.s001.tif]

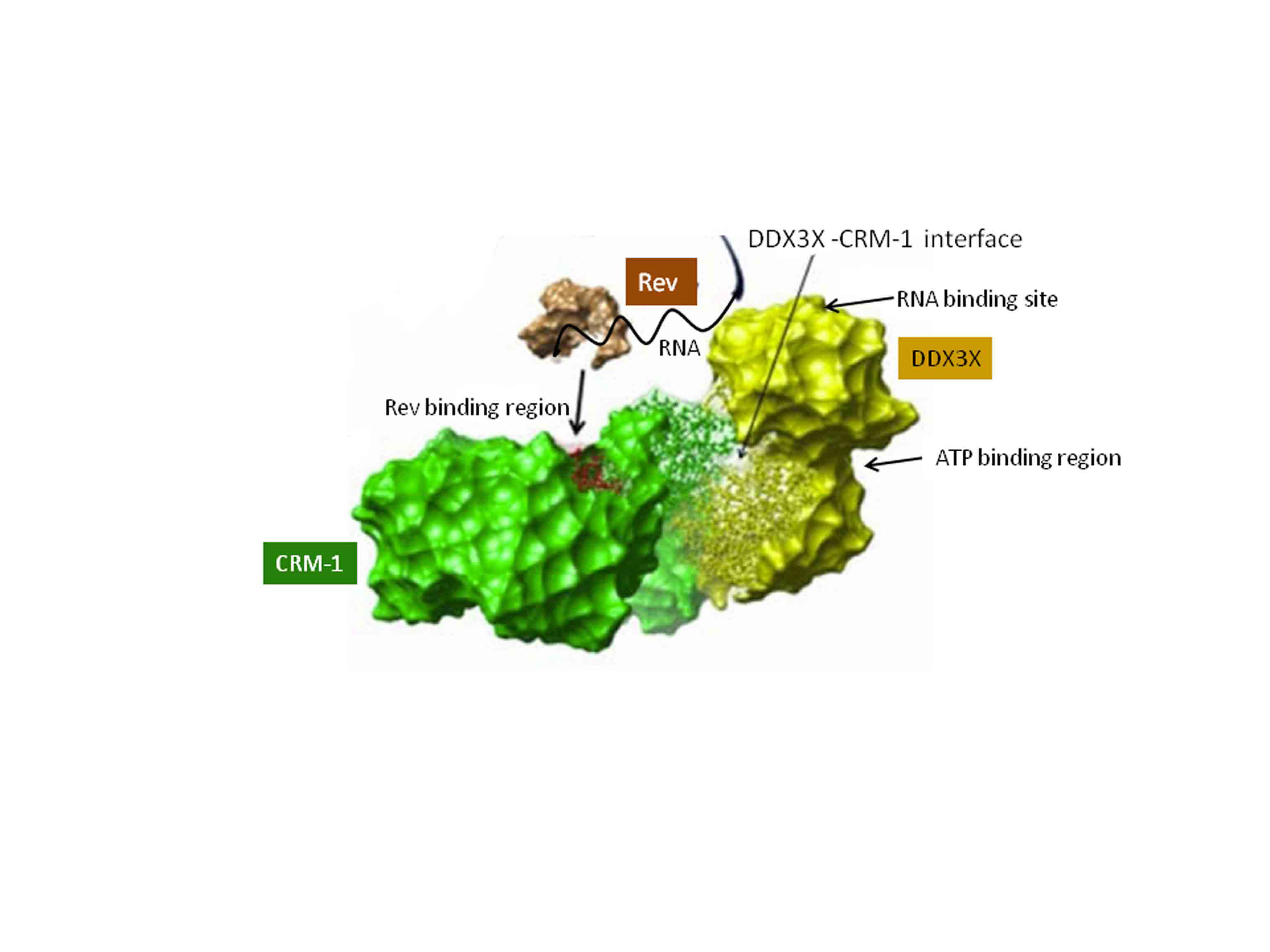

Supplement: Figure S2 — Predicted association of DDX3X, CRM-1 and HIV-1 Rev. DDX3X docking with CRM-1 was found to orient DDX3X helicase domain in proximity to HIV-1 Rev that binds CRM-1 at its residue position 800 to 820. (1.56 MB TIF) [file pone.0009613.s002.tif]
