# Supplementary material for: Evolutionary Constraints Acting on DDX3X Protein Potentially Interferes with Rev-Mediated Nuclear Export of HIV-1 RNA
Source: PLoS One. 2010 Mar 15;5(3):e9613. doi: 10.1371/journal.pone.0009613 (PMC2837722; doi:10.1371/journal.pone.0009613)
Supplement: Table S1 — Stepwise docking simulations for DDX3X-CRM-1 interaction. (0.03 MB PDF) [file pone.0009613.s003.pdf]

**TABLE S1:** Stepwise docking simulations for DDX3X-CRM-1 interaction.

| Rank | Solution Number | Global Energy | Attractive VdW | Repulsive VdW | ACE | HB |
|---|---|---|---|---|---|---|
| 1 | 229 | -31.28 | -24.22 | 14.37 | -7.00 | -1.17 |
| 2 | 323 | -21.85 | -21.60 | 9.28 | 2.67 | -1.91 |
| 3 | 184 | -18.37 | -33.72 | 21.57 | 7.11 | -4.35 |
| 4 | 167 | -18.10 | -24.15 | 18.39 | 8.21 | -1.99 |
| 5 | 11 | -17.38 | -31.82 | 17.22 | 2.49 | -2.30 |
| 6 | 341 | -15.02 | -19.40 | 8.74 | 2.72 | -2.76 |
| 7 | 436 | -13.50 | -37.59 | 26.99 | 5.95 | -3.19 |
| 8 | 437 | -13.34 | -25.59 | 9.26 | 6.68 | -1.78 |
| 9 | 260 | -12.86 | -34.94 | 13.11 | 18.57 | -7.06 |
| 10 | 152 | -12.10 | -31.24 | 18.96 | 11.94 | -3.27 |
| 11 | 334 | -12.08 | -17.80 | 4.47 | 2.97 | -3.61 |

| Solution No | Score | Area | ACE | Transformation |
|---|---|---|---|---|
| 1 | 17206 | 2686.00 | 288.52 | -1.20 1.09 2.56 4.98 160.55 -6.24 |
| 2 | 16856 | 4283.80 | 126.91 | -2.55 -1.19 1.07 -10.85 73.54 44.37 |
| 3 | 16300 | 2770.70 | 359.85 | 0.03 -0.93 -0.15 -77.93 132.05 57.65 |
| 4 | 16014 | 3230.80 | 144.98 | -0.51 -1.10 -2.09 26.21 101.09 47.46 |
| 5 | 15726 | 2386.20 | 309.05 | -0.63 0.18 1.50 39.91 121.60 5.27 |
| 6 | 15422 | 2923.00 | 440.90 | 1.46 0.10 0.08 -25.47 103.53 57.70 |
| 7 | 15130 | 2334.90 | -22.58 | 2.53 -0.43 -1.07 -30.91 61.84 60.60 |
| 8 | 15024 | 2002.10 | 425.51 | -0.82 -0.59 3.06 13.61 77.95 -15.96 |
| 9 | 14698 | 1949.60 | 268.94 | -2.24 -1.11 1.52 53.64 134.97 49.15 |
| 10 | 14532 | 2193.80 | 309.15 | 1.15 0.10 -1.72 81.04 133.72 51.83 |
| 11 | 14492 | 2248.90 | -75.41 | 1.35 1.36 0.72 -78.72 78.75 23.03 |
| 12 | 14484 | 2359.10 | 324.60 | -1.40 -0.54 -0.92 -81.06 108.52 8.79 |
| 13 | 14146 | 2283.50 | 321.81 | -1.18 0.94 2.52 12.18 156.16 -12.06 |
| 14 | 14054 | 2283.50 | 424.53 | -2.25 0.37 -1.95 -58.88 86.76 72.92 |
| 15 | 13970 | 2108.90 | 300.93 | 0.71 0.78 -3.07 6.03 121.69 17.81 |

| Rank | Solution No. | Global Energy | Attractive VdW | Repulsive VdW | ACE | HB |
|---|---|---|---|---|---|---|
| 1 | 1 | -33.37 | -38.36 | 29.92 | 8.45 | -4.14 |
| 2 | 2 | -30.18 | -26.82 | 13.16 | -6.33 | -1.70 |
| 3 | 11 | -24.54 | -44.20 | 19.02 | 16.76 | -5.68 |
| 4 | 4 | -21.12 | -43.69 | 26.38 | 7.49 | -1.67 |
| 5 | 5 | -18.91 | -34.77 | 14.91 | 12.51 | -3.30 |
